# Primary Repair of Oesophageal Perforation, An experience of 54 cases

**DOI:** 10.1186/1749-8090-10-S1-A17

**Published:** 2015-12-16

**Authors:** Amer Bilal

**Affiliations:** 1 Dept of Cardiothoracic Surgery, Lady Reading Hospital, Peshawar, Pakistan

## Background/Introduction

Esophageal perforation is associated with high morbidity and mortality rates, therefore prompt diagnosis and effective treatment are important for thoracic esophageal perforations. Primary repair provides good results for repair of thoracic esophageal perforations

## Aims/Objectives

To assess the outcome of primary repair of oesophageal perforation

## Method

54 patients who underwent primary repair of Oesophageal perforation from June 2002 to May 2014 were retrospectively analyzed. Patients of all ages, both sexes and benign thoracic oesophageal perforation were included. Malignant oesophageal perforation, benign cervical and abdominal esophageal perforation cases were excluded from the study. Patients were admitted through emergency department as a referred case after 12 hr of incident. Immediate management was resuscitation and chest intubation, kept in ICU. Contrast study was done after stabilization usually after one week. Procedure includes separate closure of mucosal and muscle layer by continuous suturing after refreshing the margins and buttressing the anastomotic area with intercostal muscle flap, followed by feeding jejunostomy. Feeding through jejunostomy tube started on second post-operative day, while contrast study was done on 7th post-operative day. Six months follow- up was done in all cases. Variable measured was postoperative leakage, stricture formation, morbidity and mortality.

## Results

Out of 54 patients, male to female ratio was 2:1, age ranges from12 to 65 years with a median age of 38 years. Perforation was caused by iatrogenic instrumentation in 45 patients, trauma in 6 and ingested foreign bodies in 4. In all patients initial chest x ray was done, location of perforation was confirmed by gastrograffin study involving upper third thoracic esophagus in 12 cases, middle third 18 and lower third in 24 cases. 7 patients developed postoperative leaks 3 patients died due to respiratory complications and 1 patient died due to myocardial Infarction. At 6 months follow-up, all 51 surviving patients were able to eat a normal diet.

## Discussion/Conclusion

Primary repair and tissue reinforcement of benign oesophageal perforation is safe in early cases and obviates the need for a second operation.

